# The Role of *PIK3CA* Mutations among Lung Adenocarcinoma Patients with Primary and Acquired Resistance to EGFR Tyrosine Kinase Inhibition

**DOI:** 10.1038/srep35249

**Published:** 2016-10-13

**Authors:** Shang-Gin Wu, Yih-Leong Chang, Chong-Jen Yu, Pan-Chyr Yang, Jin-Yuan Shih

**Affiliations:** 1 Department of Internal Medicine, National Taiwan University Hospital Yun-Lin Branch, Yun-Lin, Taiwan; 2 Graduate Institute of Clinical Medicine, College of Medicine, National Taiwan University, Taipei, Taiwan; 3 Department of Pathology, National Taiwan University Hospital, College of Medicine, National Taiwan University, Taipei, Taiwan; 4 Department of Internal Medicine, National Taiwan University Hospital, and College of Medicine, National Taiwan University, Taipei, Taiwan

## Abstract

To understand the impact of *PIK3CA* mutations on clinical characteristics and treatment response to epidermal growth factor tyrosine kinase inhibitors (EGFR TKIs) of lung adenocarcinoma, we examined *PIK3CA* and *EGFR* mutations in lung adenocarcinoma patients, and analyzed their clinical outcomes. Surgically excised tumor, bronchoscopy biopsy/brushing specimens and pleural effusions were prospectively collected from 1029 patients. *PIK3CA* and *EGFR* mutations were analyzed by RT-PCR and direct sequencing. In EGFR TKI-nave specimens, *PIK3CA* mutation rate was 1.8% (14/760). Twelve patients had coexisting *PIK3CA* and *EGFR* mutations. Among the 344 EGFR TKI-treated *EGFR* mutant patients, there was no significant difference in treatment response (*p* = 0.476) and progression-free survival (*p* = 0.401) of EGFR TKI between *PIK3CA* mutation-positive and negative patients. The *PIK3CA* mutation rate in lung adenocarcinoma with acquired resistance to EGFR TKI is not higher than that in EGFR TKI-naïve tissue specimens (2.9% (6/207) vs. 1.8%; *p* = 0.344). Of the 74 patients with paired specimens (TKI-naïve and acquired resistance to TKIs) only one patient (1.4%) developed acquired *PIK3CA* (E545K) mutation, and he also had acquired *EGFR* (T790M) mutation. In conclusion, *PIK3CA* mutation may not be associated with primary resistance to EGFR TKI among lung adenocarcinoma patients. Acquired *PIK3CA* mutation related to EGFR TKI treatment is rare.

The research of novel cancer driver genes and associated pathways established the molecular-targeted medications and the era of personalized medication. For example, epidermal growth factor receptor (EGFR)-tyrosine kinase inhibitors (TKIs) often are highly effective in lung cancer patients with somatic *EGFR* mutation^1^,^2^. Driver mutations may be the key in determining the response to target therapy.

The phosphoinositide 3-kinases (PI3Ks) constitute a lipid kinase family, and they are responsible for coordinating a diverse range of cell functions including proliferation, cell survival, degranulation, vesicular trafficking and cell migration^3^. The *PIK3CA* gene has been found in high frequencies in colon cancer (32%), glioblastoma (27%) and gastric cancer (25%)^4^. *PIK3CA* signaling pathway alterations and the frequencies of *PIK3CA* mutations were varied in different types of non-small cell lung cancer (NSCLC) in different published studies^4^,^5^,^6^,^7^. PIK3 inhibitors also render preliminary antitumor activity in preclinical studies and early phase clinical trials^8^,^9^.

PI3K and Akt are part of an important signaling pathway downstream from EGFR. PI3K/AKT pathway is important in the oncogenesis and progression of lung cancer^10^,^11^. *PIK3CA* mutation has been reported to have correlation with poor survival of NSCLC patients^7^. In a preclinical study, introduction of an activated *PIK3CA* c.1633G > A (p.E545K) mutation into the *EGFR* mutation positive cell line, HCC827, confers resistance to gefitinib^12^. Prior studies also showed that *PIK3CA* mutation is a predictor for resistance to EGFR TKIs^7^,^13^. However, these studies had relatively small number of patients. Whether the co-existing *PIK3CA* mutations cause primary resistance to EGFR TKI in lung adenocarcinoma was still not well studied.

Although having *EGFR* mutations could predict a favorable EGFR TKI treatment response, acquired resistance develops eventually. Secondary *EGFR* c.2369C > T (p.T790M) mutation is detected in 50–60% of lung adenocarcinoma patients after acquiring resistance to EGFR TKIs. In about 5% of the patients with acquired resistance to EGFR TKIs, *PIK3CA* mutations had also been reported to play a role^13^,^14^. However, Yu *et al*. did not detect any acquired *PIK3CA* mutations in their study of 88 patients with acquired resistance to EGFR TKI^15^. The role of *PIK3CA* mutations in acquired EGFR TKI resistance needs to be clarified.

Most driver mutations were mutually exclusive, but *PIK3CA* mutations frequently coexist with other mutations^16^,^17^. Interactions between *PIK3CA* and *EGFR* mutations are not clear. To understand the impact of *PIK3CA* mutation on clinical characteristics of advanced lung adenocarcinoma and the treatment response of EGFR TKIs, we examined *PIK3CA* and *EGFR* mutations from lung adenocarcinoma patients, and analyzed their clinical treatment outcomes.

## Results

### Tissue specimen collection

During June 2005 to July 2014, we consecutively collected 1668 tissue specimens from 1068 lung adenocarcinoma patients. The tissue specimens included 171 surgical resected tumors, 53 bronchoscopy biopsy tissue samples, 42 bronchoscopy brushing specimens and 1402 malignant pleural effusions (MPEs). Of the 1068 patients, there were 1029 patients who had adequate tissue for both *EGFR* and *PIK3CA* mutation analysis (Supplemental Table 1). A portion of the tumor samples was analyzed previously for *EGFR* mutation studies^18^.

**Figure 1:**
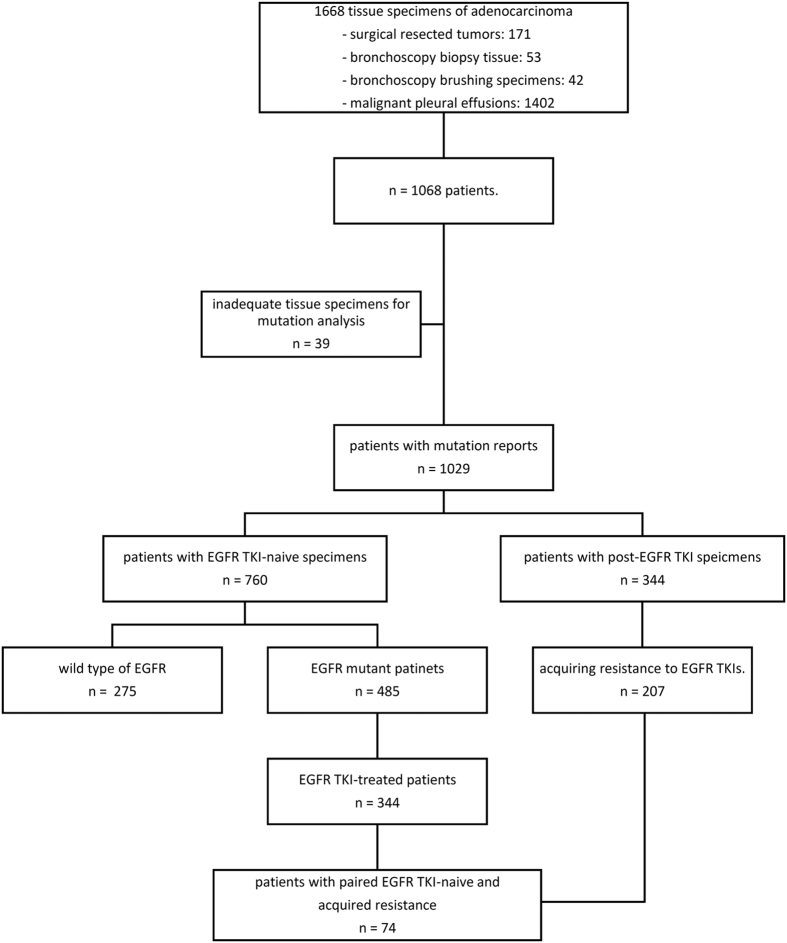
Patient selection flow chart.

Among the 1029 patients, there were 538 females (52.3%) and 730 never-smokers (70.9%). The median age was 65.4 years (range, 26.8–95.5 years). The clinical characteristics of these patients are presented in Supplemental Table 2. There were 344 patients who had tissue specimens collected after EGFR TKI treatment and 760 patients who had EGFR TKI-naїve tissue specimens (Fig. 1).

Among the 344 patients with post-TKI tissue specimens, 207 had acquired resistance to EGFR TKIs. Only 74 patients had adequate paired tissue specimens of EGFR TKI-naïve and acquired resistance to EGFR TKI for *EGFR* and *PIK3CA* mutation analysis (Supplemental Tables 2 and 3).

### Clinical characteristics of the EGFR TKI-naïve patients of lung adenocarcinoma

Among the 760 patients with EGFR TKI-naïve tissue specimens, there were 382 (50.3%) females and 532 (70.0%) never-smokers. The median age was 66.3 years (range, 26.8–95.5 years). The clinical characteristics of these patients are presented in Table 1. There were 485 (63.8%) *EGFR* mutations and 14 (1.8%) *PIK3CA* mutations. The *PIK3CA* mutation types included 2 c.1624G > A (p.E542K), 7 c.1633G > A (p.E545K), 1 c.1633G > C (p.E545Q), and 4 c.3140A > G (p.H1047R). The *PIK3CA-*mutant tissue specimens came from 4 surgical excision tumors (4 of 165; 2.4%), 1 CT-guided biopsy tissue (1 of 39; 2.6%), 2 bronchoscopy brushing specimens (2 of 34; 5.9%) and 7 MPEs (7 of 522; 1.3%). There were no differences between *PIK3CA* mutation and tissue specimen sources (*p* = 0.239).

No obvious clinical characteristics correlated with *PIK3CA* mutations except tumor staging at initial diagnosis (Table 1). *PIK3CA* mutation-positive patients were at earlier cancer stage at initial diagnosis than *PIK3CA* mutation-negative patients. (*p* = 0.048). Twelve of 14 *PIK3CA* mutation-positive patients (85.7%) had coexisting *EGFR* mutations, including: 6 with deletions in exon-19, 4 c.2573T > G (p.L858R), one c.2156G > C (p.G719A) and one p.D770 > GY. Deletions in exon-19 and c.2573T > G (p.L858R) are termed ‘classical’ activating *EGFR* mutations, which are associated with good treatment response to EGFR-TKI^2^. Patients with c.2156G > C (p.G719A) mutation also have moderate EGFR TKI sensitivity^19^. NSCLC with p.D770 > GY of exon 20 mutation leads to resistance to EGFR TKI treatment^20^.

### EGFR TKI treatment response and PFS in *EGFR* mutant lung adenocarcinoma

To evaluate the impact of *PIK3CA* mutations on EGFR TKI treatment responses, we focused on the 344 EGFR TKI-treated EGFR mutant lung adenocarcinoma patients with EGFR TKI-naïve tissue specimens (Fig. 1).

Patients’ EGFR TKI treatments included 223 gefitinib, 107 erlotinib and 14 afatinib. Their maximum treatment response of EGFR TKIs were 270 (78.5%) partial response (PR), 19 (5.5%) stable disease (SD) and 55 (16.0%) progressive disease (PD). The median progression free survival was 8.8 months (95% confidence interval: 7.9 – 9.7 months).

Of the 344 *EGFR* mutation-positive patients, there were 6 *PIK3CA* mutation-positive patients. There were no significant differences in clinical characteristics between patients with and without *PIK3CA* mutations (Table 2). Their maximum treatment responses to EGFR TKI were 2 PR, one SD and one PD. There were no significant differences in EGFR TKI response between *PIK3CA* mutation and *PIK3CA* wild type patients (*p* = 0.476). The patient with PD had a coexisting insensitive *EGFR* mutation in exon 20, p.D770 > GY.

We also analyzed the 344 EGFR TKI-treated patients’ clinical characteristics according to the three EGFR TKI agents (Supplemental Table 3). Patients who took afatinib were younger than those who received gefitinib or erlotinib treatment (*p* = 0.043). Gefitinib-treated patients had more female than the erlotinib-or afatinib-treated patients (*p* = 0.003). There was no difference in smoking history, performance status, *EGFR* mutation types, EGFR TKI treatment response and *PIK3CA* mutation among the three groups.

**Figure 2:**
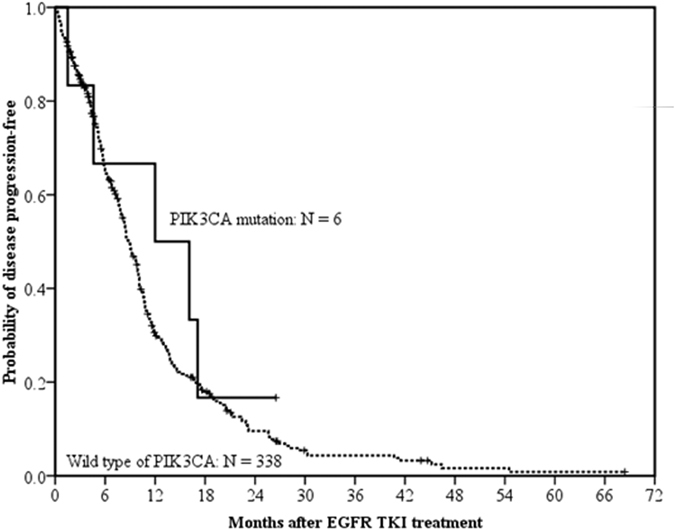
Progression-free survival of EGFR TKI treatment in EGFR mutation positive patients with EGFR TKI-naïve tissue specimens. The difference in progression-free survival of EGFR TKIs between patients with *PIK3CA* mutations (solid line, n = 6) and those with wild type of *PIK3CA* (dashed line, n = 338) did not reach statistical significance (median, 12.0 months vs. 8.8 months; *p* = 0.401, by the log-rank test).

**Figure 3:**
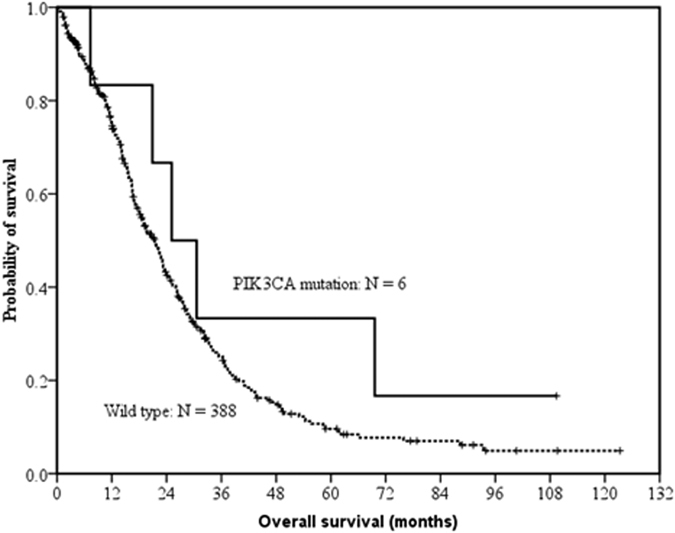
Kaplan–Meier curve of overall survival (OS) in EGFR mutant patients with EGFR TKI-naïve tissue specimens. The difference in OS between patients with *PIK3CA* mutations (solid line, n = 6) and those with wild type *PIK3CA* (dashed line, n = 338) did not reach statistical significance (median, 25.1 months vs. 21.4 months; *p* = 0.247, by the log-rank test).

The difference in PFS of EGFR TKIs between *PIK3CA* mutation-positive patients (n = 6) and those with wild type *PIK3CA* (n = 338) were not statistically significant (median, 12.0 months vs. 8.8 months; *p* = 0.401 by the log-rank test) (Fig. 2). The OS also did not reach significant differences between *PIK3CA* mutation-positive patients (25.1 months) and those with wild type *PIK3CA* (21.4 months; *p* = 0.247) (Fig. 3).

### Clinical characteristics of lung adenocarcinoma patients of acquired resistance to EGFR TKI

There were 207 patients who had tissue specimens available after acquiring resistance to EGFR TKIs. There were 128 (61.8%) females and 168 (81.2%) never-smokers. The median age was 62.3 years (range, 29.5–90.7 years). 101 (48.8%) patients had acquired *EGFR* c.2369C > T (p.T790M) mutations. The clinical characteristics of these patients are presented in Table 3. Among these, 6 patients (6 of 207; 2.9%) had *PIK3CA* mutations. All of the 6 *PIK3CA* mutation positive patients with acquired resistance to EGFR TKIs had coexisting *EGFR* mutations, including: 2 with deletions in exon 19, 3 c.2573T > G (p.L858R) and one c.2573T > G (p.L858R) + c.2327G > A (p.R776H). The *PIK3CA* mutation types included 1 c.1624G > A (p.E542K), 3 c.1633G > A (p.E545K), 1 c.3140A > T (p.H1047L) and 1 c.3140A > G (p.H1047R). Statistically, the *PIK3CA* mutation was not associated with specific EGFR TKI agents (Table 3).

In comparison with the EGFR-TKI-naïve tissue specimens, the *PIK3CA* mutation rate in tissue specimens with acquired resistance to EGFR TKI were not statistically different (1.8% vs. 2.9%; *p* = 0.344).

### *PIK3CA* mutation in paired tissue samples of EGFR TKI-naïve and acquired resistance to EGFR TKI

There were 74 patients who had enough paired EGFR TKI-naïve and acquired EGFR TKI resistant tissue samples for *EGFR* and *PIK3CA* mutation analysis. (Supplemental Table 4). 31 of the 74 patients (41.9%) had acquired *EGFR* c.2369C > T (p.T790M). Only one patient (1.4%) had *PIK3CA* mutation alteration after acquiring TKI resistance. The initial surgically resected lung adenocarcinoma showed wild type *PIK3CA* and *EGFR* c.2573T > G (p.L858R) mutation. After using gefitinib as the first line treatment for 16.1 months, disease progressed with MPE accumulation. Cancer cells in MPE had coexisting acquired *PIK3CA* c.1633G > A (p.E545K) mutation and *EGFR* c.2369C > T (p.T790M), in addition to *EGFR* c.2573T > G (p.L858R).

## Discussion

This study showed that the *PIK3CA* mutation could be detected in a small proportion (1.8%) of lung adenocarcinomas, but with high concomitant *EGFR* mutations. *PIK3CA* mutation did not confer primary resistance to EGFR TKIs, nor was it associated with a shorter PFS. The *PIK3CA* mutation rates were similar between tissues that are EGFR TKI-naïve (1.8%) and those with acquired resistance to EGFR TKI (2.9%). According to the paired tissue specimens between EGFR TKI-naïve and acquired resistance to EGFR TKI, the acquired *PIK3CA* c.1633G > A (p.E545K) mutation can be detected in only one of 74 patients (1.4%).

It is still controversial to use *PIK3CA* mutations to predict EGFR TKI treatment response. Ludovinin *et al*. showed that 6 patients with *PIK3CA* mutation had a shorter time to tumor progression (TTP) after EGFR TKI treatment, but the EGFR TKI treatment response was not associated to *PIK3CA* mutation (*p* = 0.61)^7^. Besides, other studies showed that *PIK3CA* mutation was not associated with EGFR-TKI efficacy or shorter TTP^21^,^22^. The present study also showed that *PIK3CA* mutation had no impact on treatment response or PFS of EGFR TKI in *EGFR* mutation-positive patients.

The present study showed that a *PIK3CA* mutation positive patient had PD of EGFR TKI treatment. The patient had concomitant *EGFR* exon 20 mutation, which is associated with poor gefitinib treatment response^20^. Therefore, the difference may result from the presence of concomitant *EGFR* mutations, which is the most critical point in deciding EGFR TKI treatment response^1^. However, larger studies are necessary to clarify the issue due to small number of patients presented in previous studies^7^.

“Driver mutation” is the key for developing personalized target therapy. Most driver mutations were mutually exclusive. However, the high concomitant rate of *EGFR* and *PIK3CA* dual mutation were noted in the present study. The result was similar to prior studies^16^,^23^. In addition, the French NCI’s Lung Cancer Mutation Consortium (LCMC) collected 10000 NSCLC for analysis, and the most common co-existing mutations with other drivers in NSCLC is *PIK3CA* mutation^17^. A single tumor harboring two or more coexisting PI3K pathway mutations would suggest that there would be no selective advantage for cells bearing redundant mutations. The complex PI3K network with redundancies, additive and synergistic effects have impact on tumor growth and survival, and it may affect non-linear pathways, including: negative feedback loops and non-overlapping pathway^24^. Further studies are necessary to determine whether *PIK3CA* mutation is a redundancy mutation in addition to *EGFR* mutation.

Although the dramatic treatment response was noted after target therapy treatment in tumors harboring sensitive mutations, acquired resistance develops eventually. All these acquired resistance-associated mutations developed after patients received target therapy^25^,^26^,^27^. For example: *EGFR* c.2369C > T (p.T790M) in exon 20, a secondary *EGFR* mutation, is detected in approximately half of NSCLC patients after acquiring resistance to EGFR-TKIs^25^,^26^. Sequist *et al*. reported that *PIK3CA* mutation is also associated with acquired resistance to EGFR TKI treatment^13^. The present study also found an acquired *PIK3CA* mutation change but concomitant with *EGFR* c.2369C > T (p.T790M) mutation. Whether the acquired *PIK3CA* mutations play a role in acquired resistance to EGFR TKI needs further studies.

Routine clinical pathological examination and *EGFR* mutation analysis caused substantial attrition of tumor samples^28^. In IPASS trial, only 36% of patients had adequate tissue specimens for *EGFR* mutation testing^1^. Therefore, the residual specimens in the present study were often not enough for *PIK3CA* mutation analysis. In addition, obtaining repeat biopsy for molecular analysis when patients experience disease progression is a persistent problem. Not all patients had enough pre- and post-EGFR TKI tissue specimens for mutation analysis. Therefore, we only collected 74 patients who had adequate paired samples of EGFR TKI-naïve and acquired EGFR TKI resistant for *EGFR* and *PIK3CA* mutation analysis.

*PIK3CA* mutation rates of lung adenocarcinoma ranged from 1.5% to 7.7% in different studies^5^,^7^,^29^,^30^,^31^,^32^,^33^. The present study enrolled all Asians, and most of the patients had advanced stage lung adenocarcinoma with malignant pleural effusions. Our study showed that *PIK3CA* mutation rate was 1.8% in EGFR TKI-naïve groups and 2.9% in acquired resistance to EGFR TKI group. It is similar to prior Asian studies^31^,^33^.

Of the EGFR TKI-treated subgroup and patients with acquired resistance to treatment, we only detected 6 patients with *PIK3CA* mutants. We performed a post hoc analysis of sample size of the EGFR TKI-treated subgroup and patients with acquired resistance to treatment. Assuming the frequency of *PIK3CA* mutants is 5% and a 95% confidence interval of +/−3%^13^, we estimated that 203 patients would be needed. The sample size of 207 in the present study seemed suitable for the purpose of the study.

We performed *PIK3CA* mutation detection by RT-PCR. The test was designed to detect “targeted mutation”. Although the test could not allow the detection of all variants, our mutation detection method covered more than 90% of the *PIK3CA* mutations in all lung cancer histology of the COSMIC database. *PIK3CA* mutation types identified in the present study were similar to Chaft *et al*.’s study, which enrolled 1125 lung adenocarcinoma patients for the detection of four *PIK3CA* mutation types (c.1624G > A (p.E542K), c.1633G > A (p.E545K), c.3140A > G (p.H1047R), and c.3140A > T (p.H1047L)) by mass spectrometry–based nucleic acid assay^16^.

Cancer is one of the most prominent forms of somatic mosaicism. Next-generation sequencing with deep sequence coverage enhances sensitivity and allows for accurate low-level mosaicism that would be undetectable by conventional Sanger sequencing. However, Sanger sequencing had been used to estimate the mosaic level of *PIK3CA* mutation as low as 7% in patients with megalencephaly syndrome, caused by mutations in *PIK3CA*, *PIK3R2* and *AKT3*^34^. In addition, our study group had mixed in different extent with *EGFR* mutant cell and wild-type cells to detect limitation of RT-PCR by using isolated RNA as the template followed Sanger sequencing. We could detect as low as 3% of mutant cancer cells^35^. Furthermore, if *PIK3CA* mutation(s) caused resistance to EGFR TKI, the cancer cells with *PIK3CA* mutation would proliferates with tumor progression; therefore the population of *PIK3CA* mutant cells would increase and not be in low percentage mosaicism.

The present study has some limitations. First, the patients enrolled in the study were all Asian, a population known to have high *EGFR* mutation rate. The conclusion of the present study might not be generalizable to other racial or ethnic groups due to the ethnic uniformity of the present study population. Second, the number of *PIK3CA* mutation positive patients was too small to draw a definitive conclusion. Assuming 95% confidence interval and 80% power, nearly 9000 samples are needed for the 1.1% difference between tissue samples that are EGFR TKI-naive and EGFR TKI resistant to reach statistical significance^36^. Third, in order to improve the detection sensitivity of *PIK3CA* mutation by RT-PCR, the more comprehensive method, for example: next-generation sequence, may be the alternative choice to detect different mutation types. Fourth, we did not evaluate the expression of the downstream targets of *PIK3CA*. We could not know the regulation effects of *PIK3CA* mutations on the downstream targets. This issue should be addressed in the future studies.

In conclusion, *PIK3CA* mutation may not be associated with primary resistance to EGFR TKI in lung adenocarcinoma patients. Acquired *PIK3CA* mutation related to EGFR TKI treatment is rare.

## Methods

### Patients and sample collection

During June 2005 to April 2014, we prospectively collected surgically resected lung tumors, bronchoscopy biopsy/brushing specimens and pleural effusions via thoracentesis at the National Taiwan University Hospital (NTUH). Before tissue collection, all patients were informed and wrote informed consents for future molecular studies, which was approved by the Institutional Review Board (IRB) committees of NTUH. Tissue specimens were collected for *EGFR* and *PIK3CA* sequencing. The study protocol and methods, including the experimental analysis of *EGFR* and *PIK3CA* mutation analysis, were carried out in accordance to the guidelines approved by the ethics committees at National Taiwan University Hospital.

Lung adenocarcinoma histology was categorized according to the International Multidisciplinary Classification of Lung Adenocarcinoma criteria^37^. Cytology examinations of pleural effusions were performed to confirm malignant pleural effusions (MPEs). Positive thyroid transcription factor-1 immunohistochemical stain for biopsied tumor specimens or cell blocks of MPEs confirmed the specimens as pulmonary adenocarcinoma^37^,^38^.

All of the patients’ clinical characteristics were recorded. Never-smokers were defined as patients who had smoked <100 cigarettes in their lifetime^39^. The seventh edition of Tumor-Node-Metastasis (TNM) staging system for NSCLC staging published by the International Association for the Study of Lung Cancer (IASLC) was adopted^40^.

### Response evaluation of lung adenocarcinoma patients

For each patient, physicians performed chest radiography (CXR) every 2 to 4 weeks and a chest CT scan (including the liver and adrenal glands) every 2 to 3 months and as needed to monitor the response and progression of the disease. Unidimensional method was adopted to evaluate the measurable solid tumors according to the “Response Evaluation Criteria in Solid Tumors (RECIST) guidelines (version 1.1)”^41^. We adopted Jackman’s clinical criteria to define acquired resistance to EGFR TKI treatment^27^.

EGFR TKI was taken as a single agent every day, including: erlotinib, gefitinib and afatinib. No concurrent chemotherapy or radiotherapy for the lung tumors were given during EGFR TKI therapy.

Overall survival (OS) was defined as the period from the date of first-line systemic treatment to the date of death. Progression-free survival (PFS) was defined as the period from the date of EGFR TKI treatment initiation to the date of the first objective or clinical sign of disease progression or death.

### Collection of tissue specimens

We collected pleural effusion and centrifuged the effusion at 250× g for 10 min at 4 °C. For RNA purification, the cell pellet was submerged in RNAlater (Qiagen) for storage until isolation using TRI reagent (Molecular Research Center, Cincinnati, OH) according to the manufacturer’s instruction. RNA was extracted from frozen tissue with a Qiamp RNA Mini Kit (Qiagen, Hilden, Germany) according to the manufacturer’s protocol. We used spectrophotometry to measure the amount of extracted RNA.

### Sequencing of *PIK3CA* mutations

The helical (exon 9) and kinase domains (exon 20) of *PIK3CA* gene were amplified by reverse transcription polymerase chain reaction (RT-PCR). The RNA extracted from patients’ pleural effusions were collected for RT-PCR amplification using the QIAGEN OneStep RT-PCR kit (Qiagen, Hilden, Germany) and primers as follows: exon 9, 5′-TGGTCTGTATCCCGAGAAGC-3′ (forward) and 5′-GGCCAATCTTTTACCAAGCA-3′ (reverse); and exon 20, 5′-ACGTGTGCCATTTGTTTTGA-3′ (forward) and 5′-GGTCTTTGCCTGCTGAGAGT-3′ (reverse).

The RT-PCR conditions were based on the manufacturer’s protocol. Briefly, 50 ng of total RNA was used as template and the following components were added: (1) 10 μl 5× reaction buffer, (2) 2 μl dNTP mix (10 mM each), (3) 3 μl of 10 μM forward and reverse primer each, (4) 2 μl QIAGEN OneStep RT-PCR enzyme mix and (5) RNase-free water to reach a total volume of 50 μl. The RT-PCR reaction was initiated at 50 °C for 30 min, heated to 95 °C for 15 min, then followed by 40 cycles of denaturation at 94 °C for 1 min, annealing at 60 °C for 30 s, extension at 72 °C for 1 min, and a final extension at 72 °C for 10 min. The PCR amplicons were sequenced using the same method described for *EGFR* mutation analysis.

### Sequencing of *EGFR* mutations

The Qiagen OneStep reverse transcription polymerase chain reaction (RT-PCR) kit (Qiagen) was used to obtain cDNA from extracted RNA, and exons 18–21 of EGFR were amplified. The primers and conditions of RT-PCR have been described previously^18^. PCR amplicons were sequenced using ABI PRISM 3100 or 3700 (Applied Biosystems) in both sense and antisense directions.

### Statistical analysis

We used statistical software SPSS 17.0 (SPSS Inc., Chicago, IL) for analysis. All categorical variables were analyzed by Chi-square test, except those with an expected frequency of <5, which were analyzed by Fisher’s exact test. Nonparametric Mann-Whitney U Test was used to compare the median ages of 2 groups. Survival curves were plotted using the Kaplan–Meier method and compared between groups using the log-rank test. Two-sided *p*-values of <0.05 were considered statistically significant.

## Additional Information

**How to cite this article**: Wu, S.-G. *et al*. The Role of *PIK3CA* Mutations among Lung Adenocarcinoma Patients with Primary and Acquired Resistance to EGFR Tyrosine Kinase Inhibition. *Sci. Rep.* **6**, 35249; doi: 10.1038/srep35249 (2016).

## Supplementary Material

Supplementary Information

## Figures and Tables

**Table 1:** Clinical characteristics of EGFR TKI-naïve lung adenocarcinoma patients.

	TKI-naïve patients	*PIK3CA* mutation	Wild type	P*
Total No.	760	14 (1.8%)	746 (98.2%)	
Age, median years	66.3	69.3	66.1	0.283^a^
(range)	(26.8–95.5)	(58.2–85.9)	(26.8–95.5)	
Sex				0.984#
Female	382	7 (50.0%)	375 (50.3%)	
Male	378	7 (50.0%)	371 (49.7%)	
Smoking				0.571
Never-smokers	532	11 (78.6%)	521 (69.8%)	
Smokers	228	3 (21.4%)	225 (30.2%)	
ECOG PS				0.710
0–1	638	13 (91.7%)	625 (83.8%)	
2–4	122	1 (8.3%)	121 (16.2%)	
T¶				0.267
1	90	1 (7.1%)	89 (12.1%)	
2	212	7 (50.0%)	205 (27.9%)	
3	83	2 (14.3%)	81 (11.0%)	
4	363	4 (28.6%)	350 (48.9%)	
N¶				0.842
0	227	3 (21.4%)	224 (30.5%)	
1	68	2 (14.3%)	66 (9.0%)	
2	200	4 (28.6%)	196 (26.7%)	
3	253	5 (35.7%)	248 (33.8%)	
Stage at initial diagnosis				0.048
I-IIIa	204	7 (50.0%)	197 (26.4%)	
IIIb/IV	556	7 (50.0%)	549 (73.6%)	
*EGFR*				0.098
Wild type	275	2 (14.3%)	273 (36.6%)	
Mutation	485	12 (85.7%)	473 (63.4%)	

ECOG PS, Eastern Cooperative Oncology Group performance status; EGFR, epidermal growth factor receptor; TKI, tyrosine kinase inhibitor; *PIK3CA*, phophatidylinositol-3-kinase, catalytic, alpha.

^a^By Mann-Whitney U test.

^*^By Fisher’s exact test.

^#^By Chi-square test.

^¶^There were 12 patients who received thoracentesis for pleural effusion studies, but they did not complete tumor work-up after confirming lung adenocarcinoma related malignant pleural effusions.

**Table 2:** Clinical characteristics of EGFR TKI-treated patients with EGFR TKI-naïve tissue specimens harboring *EGFR* mutations.

	Total	*PIK3CA* mutation	Wild type	P*
Total No.	344	6	338	
Age, median years	67.1	72.2	66.8	0.240^a^
(range)	(29.5–92.1)	(67.5–76.7)	(29.5–92.1)	
Sex				0.409
Female	196	2	194	
Male	148	4	144	
Smoking				0.350
Never-smokers	272	6	266	
Smokers	72	0	72	
ECOG PS				1.000
0–1	290	5	285	
2–4	54	1	53	
*EGFR mutations*				0.852#
Del-19	152	2	150	
L858R	150	3	147	
others	42	1	41	
EGFR TKIs				0.160#
gefitinib	223	2	221	
erlotinib	107	4	103	
afatinib	14	0	14	
EGFR TKI response				0.476#
PR	270	4	266	
SD	19	1	18	
PD	55	1	54	

ECOG PS, Eastern Cooperative Oncology Group performance status; EGFR, epidermal growth factor receptor; TKI, tyrosine kinase inhibitor; *PIK3CA*, phophatidylinositol-3-kinase, catalytic, alpha; PR, partial response; SD, stable disease; PD, progressive disease.

^a^By Mann-Whitney U test.

^*^By Fisher’s exact test.

^#^By Chi-square test.

**Table 3:** Clinical characteristics of lung adenocarcinoma patients with acquired resistance to EGFR TKIs.

	Acquired resistance patients	*PIK3CA* mutation	Wild type	P*
Total No.	207	6 (2.9%)	201 (97.1%)	
Age, median years	62.3	61.6	62.3	0.967^a^
(range)	(29.5–90.7)	(52.1–76.7)	(29.5–90.7)	
Sex				0.205
Female	128	2 (33.3%)	126 (62.7%)	
Male	79	4 (66.7%)	75 (37.3%)	
Smoking				0.597
Never-smokers	168	6 (100.0%)	162 (80.6%)	
Smokers	39	0 (0.0%)	39 (19.4%)	
ECOG PS				1.000
0–1	179	6 (100.0%)	173 (86.1%)	
2–4	28	0 (0.0%)	28 (13.9%)	
Stage at initial diagnosis				0.460
I-IIIa	18	1 (16.7%)	17 (9.4%)	
IIIb/IV	169	5 (83.3%)	164 (90.6%)	
*EGFR*				1.000
Wild type	9	0 (0.0%)	9 (5.0%)	
Mutation	198	6 (100.0%)	192 (95.5%)	
EGFR TKI				0.195
gefitinib	159	3 (50.0%)	156 (77.6%)	
erlotinib	43	3 (50.0%)	40 (19.9%)	
afatinib	5	0 (0.0%)	5 (2.5%)	

ECOG PS, Eastern Cooperative Oncology Group performance status; EGFR, epidermal growth factor receptor; TKI, tyrosine kinase inhibitor; *PIK3CA*, phophatidylinositol-3-kinase, catalytic, alpha.

^a^By Mann-Whitney U test.

^*^By Fisher’s exact test.

^#^By Chi-square test.
