# Supplementary material for: The Role of PIK3CA Mutations among Lung Adenocarcinoma Patients with Primary and Acquired Resistance to EGFR Tyrosine Kinase Inhibition
Source: Sci Rep. 2016 Oct 13;6:35249. doi: 10.1038/srep35249 (PMC5062358; doi:10.1038/srep35249)
Supplement: Supplementary Information [file srep35249-s1.pdf]

Patients with Primary and Acquired Resistance to EGFR Tyrosine Kinase Inhibition

Shang-Gin Wu, M.D.[1,2]; Yih-Leong Chang, M.D.[3]; Chong-Jen Yu, M.D., Ph.D.[2,4]; Pan-Chyr Yang, M.D., Ph.D. [2,4]; Jin-Yuan Shih, M.D., Ph.D. [2,4,*]

[1] Department of Internal Medicine, National Taiwan University Hospital Yun-Lin Branch, Yun-Lin, Taiwan

[2]Graduate Institute of Clinical Medicine, College of Medicine, National Taiwan University, Taipei, Taiwan;

[3] Department of Pathology, National Taiwan University Hospital, College of Medicine, National Taiwan University, Taipei, Taiwan

[4] Department of Internal Medicine, National Taiwan University Hospital, and College of Medicine, National Taiwan University, Taipei, Taiwan

* Correspondence to: Dr. Jin-Yuan Shih, M.D. Ph.D., Department of Internal Medicine, National Taiwan University Hospital, No. 7, Chung-Shan South Road, Taipei 100, Taiwan.

Tel: 886+-2-23562905, Fax: +886-2-23582867 E-mail: jyshih@ntu.edu.tw

Supplemental Table 1 —Comparing the presence of *PIK3CA* and *EGFR* mutations.

		PIK3CA mutation analysis		
		Yes	No	Total
EGFR mutation analysis	Yes	1029	21	1050
	No	9	7	16
	Total	1038	28	1066

EGFR, epidermal growth factor receptor; *PIK3CA*, phophatidylinositol-3-kinase, catalytic, alpha.

Supplemental Table 2 — Clinical characteristics of lung adenocarcinoma patients (N=1029)

Variable		Patients (%)
**Age, years, median (range)**		65.4 (26.8–95.5)
**Sex**		
	**Female**	538 (52.3%)
	**Male**	491 (47.7%)
**Smoking**		
	**Never-smokers**	730 (70.9%)
	**Smokers**	299 (29.1%)
**ECOG PS**		
	**0-1**	877 (85.2%)
	**2-4**	152 (14.8%)
**Stage at initial diagnosis**		
	**I-IIIa**	237 (23.0%)
	**IIIb/IV**	792 (77.0%)
EGFR		
	**Wild type**	362 (35.2%)
	**Mutation**	667 (64.8%)
**Tissue specimen for *PIK3CA* mutation test**		
	**EGFR TKI-naïve**	760 (73.9%)
	**Post-TKI**	344 (33.4%)
	**Acquired resistance to TKI**	207(20.1%)

ECOG PS, Eastern Cooperative Oncology Group performance status; EGFR, epidermal growth factor receptor; TKI, tyrosine kinase inhibitor; *PIK3CA*, phophatidylinositol-3-kinase, catalytic, alpha.

Supplemental Table 3 — Clinical characteristics of EGFR TKI-treated patients with EGFR TKI-naïve tissue specimens harboring *EGFR* mutations.

	Total	*gefitinub*	erlotinib	afatinib	*P**
**Total No.**	344	223	107	14	
**Age, median years**	67.1	68.3	65.2	58.6	0.042[a]
**(range)**	(29.5–92.1)	(29.5–92.1)	(33.2-88.5)	(40.3–82.3)	
**Sex**					0.003
**Female**	196	142 (63.7%)	47 (43.9%)	7 (50.0%)	
**Male**	148	81 (36.3%)	60 (56.1%)	7 (50.0%)	
**Smoking**					0.088
**Never-smokers**	272	184 (82.5%)	77 (72.0%)	11 (78.6%)	
**Smokers**	72	39 (17.5%)	30 (28.0%)	3 (21.4%)	
**ECOG PS**					0.621
**0-1**	290	186 (83.4%)	91 (85.0%)	13 (92.9%)	
**2-4**	54	37 (16.6%)	16 (15.0%)	1 (7.1%)	
***EGFR* mutations**					0.308
**Del-19**	152	97 (43.5%)	51 (47.7%)	4 (28.6%)	
**L858R**	150	103 (46.2%)	40 (37.4%)	7 (50.0%)	
**others**	42	23 (10.3%)	16 (15.0%)	3 (21.4%)	
**EGFR TKI response**					0.231
**PR**	270	178 (79.8%)	79 (73.8%)	13 (92.9%)	
**SD**	19	13 (5.8%)	5 (4.7%)	1 (7.1%)	
**PD**	55	32 (14.3%)	23 (21.5%)	0 (0.0%)	
PIK3CA					0.160
**Mutation**	6	2 (0.9%)	4 (3.7%)	0 (0.0%)	
**Wild type**	338	221 (99.1%)	103 (96.3%)	14 (100.0%)	

ECOG PS, Eastern Cooperative Oncology Group performance status; EGFR, epidermal growth factor receptor; TKI, tyrosine kinase inhibitor; *PIK3CA*, phophatidylinositol-3-kinase, catalytic, alpha; PR, partial response; SD, stable disease; PD, progressive disease. Del-19, deletion in exon-19.

[a]By Kruskal-Wallis Test

Supplemental Table 4 — Patients who had paired malignant pleural effusions sampling of EGFR TKI-naïve and acquired resistance to EGFR TKI.

		patients
**Total No.**		74
**Age, median years**		67.5
	**(range)**	(29.5–90.2)
**Sex**		
	**Female**	43
	**Male**	31
**Smoking**		
	**Never-smokers**	62
	**Smokers**	12
**ECOG PS**		
	**0-1**	61
	**2-4**	13
**EGFR TKI used**		
	**gefitinib**	57
	**erlotinib**	17
**TKI treatment received**		
	**1st line**	49
	**2nd line**	15
	**3rd line**	7
	**4th line**	3
***EGFR* mutation**		
	**Del-19**	32
	**L858R**	37
	**Other**	5

ECOG PS, Eastern Cooperative Oncology Group performance status; EGFR, epidermal growth factor receptor; TKI, tyrosine kinase inhibitor; *PIK3CA*, phophatidylinositol-3-kinase, catalytic, alpha; Del-19, deletion in exon-19.
